# Mpox Cases Among Cisgender Women and Pregnant Persons — United States, May 11–November 7, 2022

**DOI:** 10.15585/mmwr.mm7201a2

**Published:** 2023-01-06

**Authors:** Lisa P. Oakley, Kaitlin Hufstetler, Jesse O’Shea, J. Danielle Sharpe, Cristin McArdle, Varsha Neelam, Nicole M. Roth, Emily O. Olsen, Maren Wolf, Leah Zilversmit Pao, Jeremy A. W. Gold, K. Meryl Davis, Dana Perella, Shara Epstein, Maura K. Lash, Olivia Samson, Jessica Pavlick, Amanda Feldpausch, Jennifer Wallace, Atmaram Nambiar, Van Ngo, Umme-Aiman Halai, Claudia W. Richardson, Traci Fowler, Burnestine P. Taylor, Joyce Chou, Lindsey Brandon, Rose Devasia, Erin K. Ricketts, Catherine Stockdale, Mellisa Roskosky, Rachel Ostadkar, Yeng Vang, Romeo R. Galang, Kiran Perkins, Melanie Taylor, Mary Joung Choi, Paul J. Weidle, Patrick Dawson, Sascha Ellington, CDC Mpox Analytics Team, CDC Mpox Analytics Team, Cori Dennison, Ian Hennessee, Aspen Riser, LaTweika Salmon-Trejo, Gail Scogin, Emily Sims, Penelope Strid, Raquel Velazquez-Kronen, Claire Xu, Carla Zelaya

**Affiliations:** ^1^CDC Mpox Emergency Response Team; ^2^CDC Foundation, Atlanta, Georgia; ^3^Epidemic Intelligence Service, CDC; ^4^Philadelphia Department of Public Health, Philadelphia, Pennsylvania; ^5^New York City Department of Health and Mental Hygiene, New York, New York; ^6^Georgia Department of Public Health; ^7^Pennsylvania Department of Health; ^8^Los Angeles County Department of Public Health, Los Angeles, California; ^9^Detroit Health Department, Detroit, Michigan; ^10^Michigan Department of Health & Human Services; ^11^Alabama Department of Public Health; ^12^Missouri Department of Health and Senior Services; ^13^Shelby County Health Department, Memphis, Tennessee; ^14^Tennessee Department of Health; ^15^North Carolina Department of Health and Human Services; ^16^Communicable Disease, Epidemiology and Immunizations, Public Health – Seattle & King County, Seattle, Washington; ^17^Minnesota Department of Health.; CDC; CDC; CDC; CDC; CDC; CDC; CDC; CDC; CDC; CDC.

Monkeypox (mpox) cases in the 2022 outbreak have primarily occurred among adult gay, bisexual, and other men who have sex with men (MSM); however, other populations have also been affected (*1*). To date, data on mpox in cisgender women and pregnant persons have been limited. Understanding transmission in these populations is critical for mpox prevention. In addition, among pregnant persons, *Monkeypox virus* can be transmitted to the fetus during pregnancy or to the neonate through close contact during or after birth (*2*–*5*). Adverse pregnancy outcomes, including spontaneous abortion and stillbirth, have been reported in previous mpox outbreaks (*3*). During May 11–November 7, 2022, CDC and U.S. jurisdictional health departments identified mpox in 769 cisgender women aged ≥15 years, representing 2.7% of all reported mpox cases.^†^ Among cases with available data, 44% occurred in cisgender women who were non-Hispanic Black or African American (Black), 25% who were non-Hispanic White (White), and 23% who were Hispanic or Latino (Hispanic). Among cisgender women with available data, 73% reported sexual activity or close intimate contact as the likely route of exposure, with mpox lesions most frequently reported on the legs, arms, and genitals. Twenty-three mpox cases were reported in persons who were pregnant or recently pregnant^§^; all identified as cisgender women based on the mpox case report form.^¶^ Four pregnant persons required hospitalization for mpox. Eleven pregnant persons received tecovirimat, and no adverse reactions were reported. Continued studies on mpox transmission risks in populations less commonly affected during the outbreak, including cisgender women and pregnant persons, are important to assess and understand the impact of mpox on sexual, reproductive, and overall health.

Data on confirmed and probable cases of mpox are electronically reported as part of national case surveillance through a standardized case report form or the National Notifiable Diseases Surveillance System.** Data are collected by health departments and include demographic characteristics, possible exposure routes, and signs and symptoms. CDC analyzed case report data for probable or confirmed^††^ cases among cisgender women aged ≥15 years and pregnant persons during May 11–November 7, 2022. In addition, CDC identified all persons with mpox reported to CDC through national case surveillance and clinical consultations who were pregnant or recently pregnant regardless of gender identity. Detailed data regarding maternal and neonatal outcomes were obtained through enhanced pregnancy surveillance.^§§^ Statistical analyses were conducted using SAS statistical software (version 9.4; SAS Institute) and restricted to cases with available data. This activity was reviewed by CDC and was conducted consistent with applicable federal law and CDC policy.^¶¶^

## Cases Among Cisgender Women

During May 11–November 7, 2022, a total of 769 cases of mpox among cisgender women, including 23 (3%) who were pregnant, were reported by 42 public health jurisdictions (Table 1). The median age was 32 years (IQR = 25–40 years). Among the 717 (93%) cisgender women with information on race and ethnicity, 313 (44%) were Black, 182 (25%) were White, and 167 (23%) were Hispanic. Among 463 (60%) cisgender women with information on recent sexual behaviors, 329 (71%) reported recent sexual activity or close intimate contact,*** including 296 (90%) who had recent sexual contact with a cisgender man and 18 (6%) who had recent sexual contact with a cisgender woman. Among the 18 patients who had recent sexual contact with a woman, 12 also had a recent male sex partner. Of the 73 cisgender women who had complete exposure data, 53 (73%) reported recent sexual or intimate contact as the likely route of exposure. Among the 16 who reported household contact or caregiving as the likely route of exposure, five reported recent sexual or intimate contact. Among the 378 cisgender women with available data on immunocompromising conditions, 35 (9%) reported any known immunocompromising condition other than HIV infection. Information on HIV status was available for 173 cisgender women, 13 (8%) of whom had HIV infection; no cisgender women with HIV infection were pregnant.

**TABLE 1:** Characteristics of cisgender women with mpox, by current or recent* pregnancy status — United States, May 11–November 7, 2022^†^

Characteristic (no. with available information)	No. (%)^§^ of cisgender women with mpox		
	All	Not currently or recently pregnant	Currently or recently pregnant
**Total (769)^¶^**	**769 (100.0)**	**746 (97.0)**	**23 (3.0)**
**Gender identity (769)**			
Cisgender woman	**769 (100.0)**	746 (100.0)	23 (100.0)
**Age (765)**			
Median, yrs (IQR)(range)	**32 (25–40) (15–89)**	32 (25–40) (15–89)	28.5 (23–32) (20–35)
Missing	**4**	4	0
**Race and ethnicity (717)**	**717 (100.0)**	696 (93.3)	21 (91.3)
American Indian or Alaska Native, non-Hispanic	**4 (0.6)**	4 (0.6)	0 (—)
Asian, non-Hispanic	**18 (2.5)**	18 (2.6)	0 (—)
Black or African American, non-Hispanic	**313 (43.7)**	298 (42.8)	15 (71.4)
Hispanic or Latino	**167 (23.3)**	165 (23.7)	2 (9.5)
Native Hawaiian or other Pacific Islander, non-Hispanic	**1 (0.1)**	1 (0.1)	0 (—)
White, non-Hispanic	**182 (25.4)**	179 (25.7)	3 (14.3)
Multiple races, non-Hispanic	**10 (1.4)**	10 (1.4)	0 (—)
Other race, non-Hispanic	**22 (3.1)**	21 (3.0)	1 (4.8)
Missing	**52**	50	2
Recent sexual partners (463)**	**463 (100.0)**	446 (60.0)	17 (73.9)
Had a recent sexual partner	**329 (71.1)**	317 (71.1)	12 (70.6)
**Gender of recent sexual partners** **^††^**			
Cisgender man	**296 (90.0)**	284 (89.6)	12 (100.0)
Cisgender woman	**18 (5.5)**	18 (5.7)	0 (—)
Transgender man	**2 (0.6)**	2 (0.6)	0 (—)
Transgender woman	**0 (—)**	0 (—)	0 (—)
Other gender identity	**2 (0.6)**	2 (0.6)	0 (—)
**Exposure (73)** ^§§^	**73 (100.0)**	61 (8.2)	12 (52.2)
Sexual or intimate	**53 (72.6)**	44 (72.1)	9 (75.0)
Household or caregiving	**16 (21.9)**	13 (21.3)	3 (25.0)
Shared food, utensils, or dishes	**12 (18.2)**	12 (19.7)	0 (—)
Shared towels or bedding	**16 (24.2)**	16 (26.2)	0 (—)
Shared transportation	**10 (15.2)**	10 (16.4)	0 (—)
Face-to-face	**14 (21.2)**	14 (23.0)	0 (—)
Shared bathrooms	**15 (22.7)**	15 (24.6)	0 (—)
Other	**16 (24.2)**	16 (26.2)	0 (—)
**Health conditions**			
Immunocompromised (excluding HIV) (378)^¶¶^	**35 (9.3)**	34 (9.3)***	1 (7.1)^†††^
HIV infection (173)	**13 (7.5)**	13 (8.1)^§§§^	0 (—)^¶¶¶^

**Abbreviation:** mpox = monkeypox.

* Recently pregnant persons had confirmed or probable mpox infection within 21 days of delivery.

^† ^Data as of November 9, 2022.

^§^ Percentages calculated using nonmissing data.

^¶^ Information on pregnant and recently pregnant persons was gathered by the national surveillance form, clinical consultation, and enhanced pregnancy surveillance.

** Sexual partner during the 3 weeks before symptom onset.

^††^ Percentage of those who had a sexual partner during the 3 weeks before symptom onset.

^§§^ Exposures are not mutually exclusive.

^¶¶^ Immunocompromising conditions include diseases (e.g., diabetes, lupus, organ transplants, stem cell transplants, and cancer) or certain medicines (e.g., chemotherapy, biologic therapies, and steroids).

*** Percentage among the 364 cisgender women who are not currently or recently pregnant with data available on any known immunocompromising conditions.

^†††^ Percentage among the 14 pregnant or recently pregnant persons with data available on any known immunocompromising conditions.

^§§§^ Percentage among the 161 cisgender women who are not currently or recently pregnant with data available on HIV status.

^¶¶¶^ Percentage among the 12 pregnant or recently pregnant persons with data available on HIV status.

Among all cisgender women with mpox and available data, the most frequently reported signs and symptoms were rash (93%), headache (54%), pruritis (57%), malaise (54%), fever (49%), and chills (49%) (Table 2). Among 376 (49%) cisgender women with data on rash location, rash was most frequently reported on the legs (48%), arms (47%), genitals (36%), and trunk (33%); 50 (16%) reported rash in one region, 63 (20%) in two regions, 57 (18%) in three regions, and 140 (45%) in four or more regions. Rash location was similar when comparing cisgender women who reported recent sexual exposure with those who did not.

**TABLE 2:** Signs and symptoms and rash sites among all cisgender women with mpox (N = 769) — United States, May 11–November 7, 2022*

Characteristic	Presence or absence of signs or symptoms, no. (%)^†^			No. missing (% of total sample)
	Present	Absent	Unknown	
**Sign or symptom**				
Rash	376 (92.6)	17 (4.2)	13 (3.2)	363 (47.2)
Headache	182 (54.3)	122 (36.4)	31 (9.3)	434 (56.4)
Malaise	176 (53.5)	115 (35.0)	38 (11.6)	440 (57.2)
Fever	165 (48.5)	139 (40.9)	36 (10.6)	429 (55.8)
Chills	162 (49.2)	135 (41.0)	32 (9.7)	440 (57.2)
Pruritis	164 (56.8)	91 (31.5)	34 (11.8)	480 (62.4)
Myalgia	142 (45.1)	142 (45.1)	31 (9.8)	454 (59.0)
Enlarged lymph nodes	137 (41.8)	153 (46.7)	38 (11.6)	441 (57.3)
Vomiting or nausea	63 (24.3)	161 (62.2)	35 (13.5)	510 (66.3)
Abdominal pain	57 (19.2)	198 (66.7)	42 (14.1)	472 (61.4)
Rectal pain	39 (12.8)	229 (74.8)	38 (12.4)	463 (60.2)
Tenesmus	22 (8.5)	199 (76.8)	38 (14.7)	510 (66.3)
Conjunctivitis	14 (5.7)	199 (80.2)	35 (14.1)	521 (67.8)
Pus or blood in stools	12 (4.6)	210 (80.5)	39 (14.9)	508 (66.1)
Rectal bleeding	11 (3.7)	246 (82.8)	40 (13.5)	472 (61.4)
Proctitis	8 (3.3)	185 (76.5)	49 (20.3)	527 (68.5)
**Rash site** ^§^				
Legs	182 (48.4)	NA	NA	NA
Arms	178 (47.3)			
Trunk	125 (33.2)			
Genitals	137 (36.4)			
Face	116 (30.9)			
Palms	81 (21.5)			
Head	79 (21.0)			
Hand	73 (19.4)			
Neck	54 (14.4)			
Mouth	57 (15.2)			
Perianal	53 (14.1)			
Soles	44 (11.7)			
Other	100 (26.6)			

**Abbreviation:** NA = not applicable.

* Data as of November 9, 2022.

^†^ Percentages calculated using nonmissing data.

^§^ Percentages among the 376 cisgender women who reported experiencing rash.

## Cases in Currently and Recently Pregnant Persons

During May 11–November 7, 2022, 23 cases of mpox were reported during pregnancy (21) or within 3 weeks of pregnancy (two); all pregnant and recently pregnant persons with mpox identified as cisgender women on the mpox case report form^†††^ (Table 1). Among 12 currently or recently pregnant persons with available exposure data, nine reported sexual contact and three reported household contact. Among 10 cases in pregnant persons with information on trimester of infection, three occurred during the first, four during the second, and three during the third trimester (Table 3). Rash was present in all persons. Genital lesions were reported by four currently or recently pregnant persons; none reported genital lesions near the time of delivery.

**TABLE 3:** Characteristics of currently and recently pregnant persons* with mpox (N = 23) — United States, May 11–November 7, 2022

Characteristic	No. (%)^†^
Currently pregnant	21 (91.3)
Recently pregnant	2 (8.7)
**Pregnancy trimester when *Monkeypox virus* infection occurred (10)**	
First	3 (30.0)
Second	4 (40.0)
Third	3 (30.0)
Missing	11
**Sign or symptom** ^§^	
Fever	6 (26.1)
Rash	23 (100.0)
Genital or breast lesions	4 (17.4)
Myalgia	2 (8.7)
Pruritis	6 (26.1)
Lymphadenopathy	3 (13.0)
**Disease severity**	
**Hospitalized**	
Yes	4 (17.4)
No	19 (82.6)
Admitted to ICU	0 (—)
**Mpox-directed therapy** ^¶^	
Tecovirimat (oral or IV)	11 (47.8)
VIGIV	0 (—)
**Postexposure prophylaxis**	
Received JYNNEOS	0 (—)

**Abbreviations:** ICU = intensive care unit; IV = intravenous; mpox = monkeypox; VIGIV= vaccinia immune globulin intravenous.

* Recently pregnant persons had confirmed or probable *Monkeypox virus* infection within 21 days of delivery.

^†^ Percentages calculated among nonmissing values.

^§^ Signs and symptoms are not mutually exclusive.

^¶^ Two women who received tecovirimat also received other treatments (e.g., acyclovir or antibiotics).

Eleven (48%) pregnant persons received tecovirimat (administered during all trimesters of pregnancy); no medication-related adverse events were reported. Four pregnant persons were hospitalized related to symptoms from *Monkeypox virus* infection (pain control and treatment of superimposed cellulitis) and remained pregnant at discharge. No pregnant person required intensive care, intubation, or unplanned delivery. None of the pregnant persons received vaccinia immune globulin intravenous (VIGIV) for treatment. Of the 21 persons who received an mpox diagnosis during pregnancy, three have reported outcomes, including two full-term deliveries without complications (including no transmission to the infant) and one spontaneous abortion at 11 weeks’ gestation. Two pregnant persons experienced mpox symptoms within 3 days after delivery; their newborns developed lesions within 1 week of their symptom onset. Both newborns received oral tecovirimat within 48 hours of developing lesions and were treated for 10–14 days; one received VIGIV. Both newborns responded to treatment, appeared to be in good health, and were discharged home.

One recently pregnant person who was breastfeeding developed lesions 4 days postpartum, including under the breast; this person’s newborn developed symptoms with lesions on the face and chest 6 days later. Two other cisgender women who were not pregnant or recently pregnant were breastfeeding at the time of mpox diagnosis. One woman’s infant was exposed to a symptomatic household contact; she experienced symptoms 2 weeks after the infant’s diagnosis. The second woman received an mpox diagnosis following an occupational exposure; breast milk samples from this person were tested and were negative for *Monkeypox virus* DNA by polymerase chain reaction testing.^§§§^

## Discussion

*Monkeypox virus* infections in cisgender women during May 11–November 7 constituted <3% of total U.S. cases. Consistent with disparities observed overall during the ongoing mpox epidemic, the proportion of Black and Hispanic women with mpox was higher than the proportion of Black and Hispanic women in the U.S. population (*6*). This finding is similar to disparities among mpox cases in the United States overall and underscores the continued need for public health efforts to provide education on prevention of mpox and ensure equitable access to mpox vaccination, testing, and treatment.

Sex or close intimate contact within 3 weeks of symptom onset was reported by nearly three quarters of cisgender women with mpox, and genital lesions were frequently reported, suggesting sexual exposure as a likely primary route of transmission. Obstetrician-gynecologists and other providers should consider mpox when examining new genital, oral, or breast lesions. Patient education regarding risks for transmission of *Monkeypox virus* and other sexually transmitted infections should be provided.

Genital lesions in pregnant persons pose a risk for *Monkeypox virus* transmission to the fetus during vaginal delivery.^¶¶¶^ A thorough skin and mucosal (e.g., anal, vaginal, and oral) examination for mpox lesions should be performed in persons with possible mpox near the time of delivery to identify lesions of which they might be unaware. When mpox lesions, including genital lesions, are present, shared decision-making should be considered when discussing route of delivery. Because there might be an increased risk for severe disease in newborns, breastfeeding should be temporarily delayed until criteria for discontinuing isolation have been met (lesions have resolved, the scabs have fallen off, and a fresh layer of intact skin has formed).**** 

Clinicians caring for cisgender women and pregnant persons should become familiar with clinical considerations for the prevention, diagnosis, and treatment of mpox^††††^ and should provide pre- and postexposure prophylaxis if indicated. Vaccination with JYNNEOS should be provided to eligible persons, including those who are pregnant or breastfeeding, and providers should discuss vaccination risks and benefits.^§§§§^

The findings in this report are subject to at least three limitations. First, data for some variables such as exposure risk and HIV status were frequently missing (≤92%). Thus, these data might not represent the characteristics of the overall sample. Second, the small sample size of currently and recently pregnant persons with mpox might limit the generalizability of outcomes. Finally, additional time is needed for pregnancy completion to describe outcomes among all cases of mpox during pregnancy.

Cases of mpox have occurred primarily among adult gay, bisexual, and other MSM during the current outbreak; however, any person, including cisgender women, can also acquire infection. Public health efforts should include more emphasis on cisgender women who might be at increased risk for exposure. In addition, although most reported cases of mpox in pregnant persons have been managed in the outpatient setting, some persons might require hospitalization, and there is a risk for perinatal transmission. To mitigate this risk, pregnant, recently pregnant, and breastfeeding persons should be offered prophylaxis or treatment if indicated. Continued collection of information is critical to evaluating the risk for transmission, informing infection prevention and control, and assessing the impact of mpox on the sexual, reproductive, and overall health of cisgender women. In addition, collection of longitudinal data among pregnant persons and their infants is critical to understanding the effects of mpox on maternal and neonatal outcomes. CDC, in collaboration with health departments, will continue to follow cases in pregnant and recently pregnant persons and provide updates as data become available.

**Summary**

**What is already known about this topic?**

Data from the ongoing monkeypox (mpox) outbreak on cases in cisgender women and in pregnancy are limited.

**What is added by this report?**

Among 769 mpox cases reported among U.S. cisgender women, Black or African American and Hispanic or Latino women were disproportionately affected. Most cisgender women reported recent sexual activity with men. Twenty-three cases among pregnant or recently pregnant persons were reported and all recovered. Four pregnant persons were hospitalized for mpox, and tecovirimat was tolerated with no adverse reactions.

**What are the implications for public health practice?**

Continued monitoring of mpox risk in cisgender women and during pregnancy is critical to assessing the impacts of mpox on sexual, reproductive, and overall health and to better understand perinatal outcomes.

## Footnotes

* These authors contributed equally to this report.

† https://www.cdc.gov/poxvirus/monkeypox/response/2022/demographics.html (Accessed November 7, 2022).

§ Recently pregnant persons had confirmed or probable *Monkeypox virus* infection within 21 days of delivery.

¶
https://www.cdc.gov/poxvirus/monkeypox/health-departments/case-reporting.html

**
https://www.cdc.gov/nndss/index.html

††
https://www.cdc.gov/poxvirus/monkeypox/clinicians/case-definition.html

§§ Enhanced pregnancy surveillance data were collected through direct communication with jurisdictions, under an Assurance of Confidentiality, leveraging existing mother-baby linked surveillance. https://www.cdc.gov/ncbddd/set-net/index.html

¶¶ 45 C.F.R. part 46.102(l)(2), 21 C.F.R. part 56; 42 U.S.C. Sect. 241(d); 5 U.S.C. Sect. 552a; 44 U.S.C. Sect. 3501 et seq.

*** Defined as any sex or close intimate contact during the 3 weeks before symptom onset.

††† CDC recognizes that not all pregnant persons are cisgender women. However, in the study, all cases of mpox in pregnant persons, recently pregnant persons, and breastfeeding persons occurred in persons who identified as cisgender women based on the mpox case report form.
